# Time-resolved structural studies of protein reaction dynamics: a smorgasbord of X-ray approaches

**DOI:** 10.1107/S0108767309054361

**Published:** 2010-02-18

**Authors:** Sebastian Westenhoff, Elena Nazarenko, Erik Malmerberg, Jan Davidsson, Gergely Katona, Richard Neutze

**Affiliations:** a Department of Chemistry, Biochemistry and Biophysics, University of Gothenburg, Box 462, SE-40530 Gothenburg, Sweden; b Department of Photochemistry and Molecular Science, Uppsala University, Box 523, SE-75120 Uppsala, Sweden

**Keywords:** time-resolved diffraction, structural biology, protein structural dynamics, Laue diffraction, kinetic crystallography, WAXS, XAS

## Abstract

Proteins undergo conformational changes during their biological function. As such, a high-resolution structure of a protein’s resting conformation provides a starting point for elucidating its reaction mechanism, but provides no direct information concerning the protein’s conformational dynamics. Several X-ray methods have been developed to elucidate those conformational changes that occur during a protein’s reaction, including time-resolved Laue diffraction and intermediate trapping studies on three-dimensional protein crystals, and time-resolved wide-angle X-ray scattering and X-ray absorption studies on proteins in the solution phase. This review emphasizes the scope and limitations of these complementary experimental approaches when seeking to understand protein conformational dynamics. These methods are illustrated using a limited set of examples including myoglobin and haemoglobin in complex with carbon monoxide, the simple light-driven proton pump bacteriorhodopsin, and the superoxide scavenger superoxide reductase. In conclusion, likely future developments of these methods at synchrotron X-ray sources and the potential impact of emerging X-ray free-electron laser facilities are speculated upon.

## Abstract 2

Time-resolved structural studies of proteins have undergone several significant developments during the last decade. Recent developments using time-resolved X-ray methods, such as time-resolved Laue diffraction, low-temperature intermediate trapping, time-resolved wide-angle X-ray scattering and time-resolved X-ray absorption spectroscopy, are reviewed.

## 1. Introduction

Structural biology is an extremely successful field of the life sciences. Currently approximately 20 new structures are deposited daily within the Protein Data Bank, adding to an accumulated total of more than 60 000 structural entries. This mass of structural knowledge has provided detailed biological insight into protein structure and function relationships and uncountable details into the specific biochemistry of cellular reactions. Despite this impressive history of success, understanding the details of protein conformational changes presents a major challenge for structural biology. This is because X-ray diffraction, by far the most successful technique for elucidating new protein structures at high resolution, intrinsically relies upon the presence of well ordered arrangements of identical copies of the same protein. Flexible regions of a protein are frequently not visible within a crystal structure and proteins known to display large levels of flexibility, such as membrane protein transporters and receptors (White, 2009 ▶), are typically very difficult to crystallize. Likewise, it is not possible even in principle to crystallize a transient protein conformation and rather one must either initiate a chemical reaction within three-dimensional protein crystals (Hajdu *et al.*, 2000 ▶; Schlichting, 2000 ▶) or stabilize a desired conformation by using point mutations, substrate analogues or inhibitors.

To directly address functional questions involving protein conformational dynamics one would ideally record a movie of these structural changes as they happen, as Muybridge famously did in 1878 when asked to settle an assertion concerning the gait of a horse while galloping (Haas, 1976 ▶). Several X-ray approaches have been developed to take on this challenge including time-resolved Laue diffraction and intermediate trapping (or so-called kinetic crystallography) studies, which are both X-ray diffraction methods and therefore rely upon well ordered three-dimensional crystals. Time-resolved wide-angle X-ray scattering from proteins has also recently emerged as a method for characterizing large-scale global conformational changes in proteins in a liquid environment, whereas time-resolved X-ray absorption studies provide structural details around metal centres in the solution phase. Each of these methods has its intrinsic advantages and disadvantages and provides a distinct, yet complementary, picture of protein conformational dynamics.

In this review we summarize all four of these X-ray methods for characterizing protein conformational changes. To illustrate the conceptual and technical issues at hand we emphasize two main biophysical problems: the structural dynamics of the soluble proteins myoglobin and haemoglobin when bound carbon monoxide is dislodged using light, and the structural mechanism of proton pumping by the integral membrane protein bacteriorhodopsin. A smaller number of other examples, in particular the superoxide scavenger superoxide reductase, are also used in places to illustrate technical points or sketch other successful applications. As such this review does not provide a comprehensive overview of the most recent and insightful structural results from within the field of time-resolved structural biology (Hajdu *et al.*, 2000 ▶; Bourgeois & Royant, 2005 ▶; Neutze *et al.*, 2002 ▶; Schlichting & Chu, 2000 ▶; Moffat, 1998*b* ▶) but rather we concentrate upon the developing methodologies *per se*: their strengths, weaknesses and the complementary nature of structural results emerging from these different techniques.

In presenting this review we first describe the method of time-resolved Laue diffraction, which allows the observation of three-dimensional structural changes in proteins in real time. Monochromatic X-ray data collection from intermediates trapped at low temperature is then introduced, which is a method that yields high-resolution three-dimensional structures but lacks the explicit time resolution of Laue diffraction. Time-resolved wide-angle X-ray scattering is presented as an emerging method which side-steps some conceptual concerns arising when working with proteins in a crystalline phase (Heberle & Gensch, 2001 ▶). Finally, time-resolved X-ray absorption spectroscopy studies of proteins are briefly reviewed, illustrating the potential and challenges of X-ray spectroscopy. These impressive advances have depended explicitly upon the availability of intense X-ray radiation at third-generation synchrotron sources. As such we close by reflecting upon possible future developments of the field using synchrotron radiation and the tremendous promise offered by highly intense X-ray free-electron lasers, from which the first experimental results are now emerging.

## 2. Time-resolved Laue diffraction

Conceptually, the definitive goal of time-resolved structural biology is simple: to structurally characterize the transient intermediate states whose populations rise and fall as the reaction evolves. Of all of the X-ray techniques at hand, it is time-resolved Laue diffraction that in principle comes closest to this goal. In this approach a reaction is initiated in three-dimensional crystals at room temperature and X-ray diffraction data are rapidly collected using a broad-bandwidth X-ray beam. Two key advantages emerge from the use of a broad spectrum X-ray beam (so-called ‘white’ wiggler; or ‘pink’ undulator spectra): the first is that the maximum available X-ray flux from the synchrotron insertion device may be utilized; the second is that the broad spectrum of the X-ray beam enables a large number of full X-ray diffraction reflections to be collected (rather than partial reflections) without the need to rotate the crystal, which is essential when one wishes to record snapshots on sub-millisecond timescales. Nevertheless, this approach encompasses a large number of technical challenges which have been widely discussed (Šrajer *et al.*, 2000 ▶; Ren *et al.*, 1999 ▶; Yang *et al.*, 1998 ▶; Bourgeois *et al.*, 1996 ▶; Moffat, 2003 ▶, 1998*a* ▶), the most limiting of which is high demands regarding low mosaic spread for the crystals of interest. Since the very structural changes that one may wish to observe can induce strain within a tightly packed crystal lattice and thereby increase crystal mosaic spread, this limitation can be both a fundamental limitation as well as a practical one.

Proof-of-principle studies of Laue diffraction from protein crystals established the principle that it was possible to record such diffraction data (Moffat *et al.*, 1984 ▶) and interpret it in terms of electron density (Hajdu *et al.*, 1987 ▶) more than two decades ago. Time-resolved Laue diffraction studies soon followed, whereby reactions were initiated within a crystal and three-dimensional electron-density snapshots extracted (Hajdu *et al.*, 1987 ▶; Schlichting *et al.*, 1990 ▶). More recent successes with time-resolved Laue diffraction have reported a steady improvement in the time resolution achieved using short-pulse laser photolysis of light-driven systems in combination with very short pulse X-ray diffraction exposures isolated at the European Synchrotron Radiation Facility (Wulff *et al.*, 1997 ▶). Short-pulse time-resolved Laue diffraction has been possible on the light-driven systems myoglobin in complex with carbon monoxide (Srajer *et al.*, 1996 ▶, 2001 ▶; Bourgeois *et al.*, 2003 ▶, 2006 ▶; Schotte *et al.*, 2003 ▶, 2004 ▶) and the light-sensor photoactive yellow protein (Genick *et al.*, 1997 ▶; Perman *et al.*, 1998 ▶; Ren *et al.*, 2001 ▶; Anderson *et al.*, 2004 ▶; Rajagopal *et al.*, 2005 ▶; Ihee, Rajagopal *et al.*, 2005 ▶; Schmidt *et al.*, 2004 ▶). These rather spectacular successes have been made possible by continuous technical improvements surrounding Laue-diffraction data collection, and their favourable diffraction properties. In particular, both systems yield crystals with very low mosaic spread that diffract to atomic resolution when monochromatic data are collected at low temperature (Vojtechovsky *et al.*, 1999 ▶; Genick *et al.*, 1998 ▶). Moreover, these crystals appear unusually resistant to radiation damage from both the X-ray probe and the intense laser pulses used to initiate their reaction cycles.

To illustrate this method we use the example of myoglobin in complex with carbon monoxide, although structural results from photoactive yellow protein could equally well have been discussed. Myoglobin is the primary oxygen-carrying pigment of muscle cells and is closely related to the multi-subunit protein haemoglobin, which transports oxygen in the blood. Myoglobin holds a special place within the field of structural biology since it was the first protein to have its structure solved by X-ray crystallography in 1958 (Kendrew *et al.*, 1958 ▶), showing that the protein contains eight α-helices which surround a single haem group. It is the conjugated double-bond system of the haem which absorbs visible light and thereby gives the protein its characteristic red colour. Because of its relative simplicity, myoglobin is commonly referred to as the hydrogen atom of biophysics. Although carbon monoxide binds to the haem group of myoglobin with a considerably higher affinity than oxygen, it can be removed by light, whereupon it rebinds again on a sub-millisecond timescale. This light-induced reversible binding can be triggered with short laser pulses and this property is exploited in time-resolved Laue diffraction studies.

**Figure 1:**
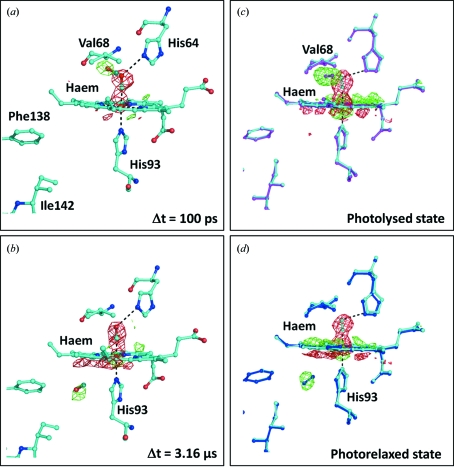
Time-resolved Laue diffraction and intermediate trapping studies of the photodissociation of carbon monoxide bound to the haem group of myoglobin. (*a*) *F*_obs_(light) − *F*_obs_(dark) difference Fourier electron density map of myoglobin (L29F) calculated for the time point 100 ps following photoactivation by a short laser pulse (Schotte *et al.*, 2003 ▶; Aranda *et al.*, 2006 ▶). Negative difference electron density (red) is observed 100 ps after photoactivation at the resting-state position of the carbon monoxide molecule (above the haem) and positive difference electron density (green) is observed nearby below Val68. (*b*) A similar map calculated for the time point 3.16 µs after photoactivation. At this time another binding pocket is visible as positive difference electron density (green) below the haem group near His93. In calculating these maps the crystallographic observations were taken from Protein Data Bank entries 2g0s (resting state), 2g0v (100 ps) and 2g14 (3.16 µs) (Aranda *et al.*, 2006 ▶). (*c*) Difference Fourier maps calculated from low-temperature trapping studies on wild-type myoglobin:carbon monoxide complexes (Chu *et al.*, 2000 ▶). The resting-state model is shown in cyan (Protein Data Bank entry 1dwr) and the photolysed state is shown in magenta (1dws). (*d*) Difference Fourier map calculated for the photorelaxed state, shown in blue (1dwt), from the same low-temperature study. All maps are contoured at 4.5σ. Agreement is apparent between the positions of the carbon monoxide molecule observed in the intermediate trapping and the Laue diffraction studies.

Fig. 1 ▶ illustrates the type of structural information accessible from a time-resolved Laue diffraction study (Schotte *et al.*, 2003 ▶; Aranda *et al.*, 2006 ▶). A strong negative difference electron density peak is visible on the carbon monoxide molecule immediately after photoactivation (Fig. 1*a* ▶, red density), indicating that this molecule is rapidly displaced from the haem iron following photodissociation. Moreover, positive difference electron density features arise within 100 ps near the original position of the carbon monoxide molecule (Fig. 1*a* ▶, green density) and another positive density feature later appears below the haem after 3.16 µs (Fig. 1*b* ▶), indicating transient binding pockets for the displaced carbon monoxide molecule. An elegant aspect of these time-resolved Laue-diffraction studies is that both the appearance and dis­appearance of carbon monoxide within a transient binding pocket can be visualized within a single experiment.

In another recent Laue-diffraction study, changes in electron density associated with the conversion from a ligated to an unligated state of the haem domain of the oxygen sensor FixL were reported (Key *et al.*, 2007 ▶). This experiment was conceptually similar to earlier work on the myoglobin:carbon monoxide complex since the protein relaxation was tracked with time following laser-induced dissociation of a carbon monoxide molecule bound to a buried haem group. Another noteworthy study applied time-resolved Laue diffraction to search for light-induced structural changes in a photosynthetic reaction centre, which marked the first application of this method to a membrane protein complex. In that case, however, no significant light-induced changes in electron density changes were reported (Baxter *et al.*, 2004 ▶).

Despite the impressive technical gains achieved over recent years, the method of time-resolved Laue diffraction has not grown as rapidly over the last two decades as other challenging fields of structural biology such as membrane protein crystallography (White, 2009 ▶). Several limitations to the method are well known and include high demands on crystal quality, the limited availability of rapid general triggering methods, and the experimentally limiting fact that most triggering methods are not reversible. As such, while providing an elegant method with high potential for unique biological insight, time-resolved Laue diffraction seems likely to remain the reserve of a small number of determined researchers rather than become a widely used tool of mainstream structural biology.

## 3. Trapped intermediate X-ray diffraction studies

Reaction cycle intermediates build up as energy barriers are encountered that must be traversed in order for the reaction to go to completion, causing the protein to ‘pause’ at particular points along the reaction coordinate. Time-resolved Laue diffraction is able to visualize the build-up of specific reaction transients at room temperature because their concentrations have accumulated to significant levels (typically 20 to 50%) at a given time delay following reaction triggering within three-dimensional crystals. An alternative approach to real-time room-temperature studies is to control the kinetics of the reaction by using temperature or the chemical environment, and thereby find conditions for which a large population of a desired intermediate becomes trapped within three-dimensional crystals. Once conditions have been identified under which the population of a specific intermediate builds up, standard monochromatic X-ray diffraction data can be collected. While conceptually less elegant than time-resolved Laue diffraction, intermediate trapping methods have proven very popular in the search to understand the structural pathways of protein-mediated reactions (Hajdu *et al.*, 2000 ▶; Bourgeois & Royant, 2005 ▶; Neutze *et al.*, 2002 ▶; Schlichting & Chu, 2000 ▶).

A great variety of different approaches have been successfully used to trap structural intermediates and intermediate state analogues in crystals for X-ray diffraction studies. For slow reactions it is relatively straightforward to initiate the reaction at room temperature and then quench it by plunging the crystal into liquid nitrogen. This has been demonstrated, for example, in studies of reaction intermediates of the ATP-dependent enzyme dethiobiotin synthetase (Kack *et al.*, 1998 ▶) and serine protease catalysis (Wilmouth *et al.*, 2001 ▶), for which reactions were triggered by chemical soaking and a pH jump, respectively. X-rays themselves have also be used to trigger a reaction at low temperature within redox-sensitive crystals (Schlichting *et al.*, 2000 ▶; Colletier *et al.*, 2008 ▶; Berglund *et al.*, 2002 ▶), which illustrates an elegant synergy between the reaction trigger and structural probe.

Optical absorption spectroscopy was the first technique that was applied to single protein crystals in order to characterize the nature and population of a specific intermediate species trapped within crystals (Hadfield & Hajdu, 1993 ▶; Rossi *et al.*, 1992 ▶). The importance of spectral characterization is that reactivity in the crystal can, and indeed should, be quantified by parallel optical studies. If reactivity in the crystal differs drastically from that in solution, optical studies will reveal it and may help the experimenter avoid conducting challenging time-resolved Laue diffraction or intermediate trapping studies under conditions for which the biological interpretation may prove controversial. While absorption spectroscopy continues to be the mainstream spectroscopic tool (McGeehan *et al.*, 2009 ▶), fluorescence and Raman spectroscopy have recently increased in popularity (McGeehan *et al.*, 2007 ▶; Bourgeois *et al.*, 2009 ▶).

**Figure 2:**
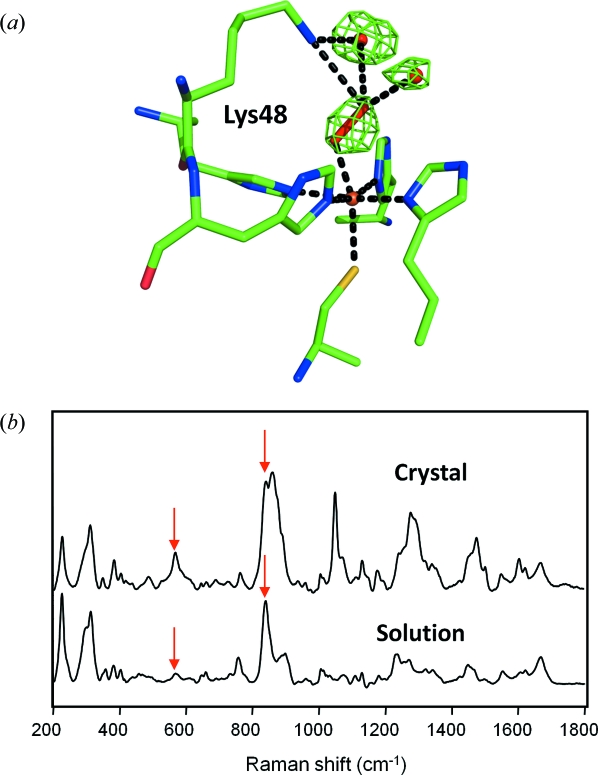
Intermediate trapping studies of the non-haem iron protein superoxide reductase from *D. barsii* (Katona *et al.*, 2007 ▶). (*a*) Structure of the iron peroxide intermediate bound in an end-on configuration in the active site of superoxide reductase. The *F*_obs_ − *F*_calc_ omit (green) maps are contoured at 4.5σ. The peroxo moiety is hydrogen-bonded to Lys48 and two water molecules of the active site, which assist the protonation *en route* to the product formation. The structural model and electron density derive from entry 2ji3 of the Protein Data Bank. (*b*) Off-resonance Raman spectra collected from single crystals and solutions of hydrogen-peroxide-treated superoxide reductase. Single crystals of superoxide reductase (top spectrum) were treated in crystallization buffer with the addition of 10 m*M* H_2_O_2_ for 3 min and subsequently flash frozen in a cryoprotected buffer. SOR solutions (bottom spectrum) were treated similarly (for details see Katona *et al.*, 2007 ▶).

To illustrate the advantages of Raman scattering when characterizing reaction species in crystals, we consider a recent intermediate trapping study of an iron peroxide intermediate in crystals of superoxide reductase (SOR) (Katona *et al.*, 2007 ▶). Superoxide reductase is a (non-haem) iron-containing protein which serves to scavenge superoxide, namely O_2_^−^ radicals which arise upon the one-electron reduction of molecular oxygen. Fig. 2(*a*) ▶ shows the electron density map recovered from superoxide reductase after crystals were treated with hydrogen peroxide for 5 min. Residual electron density (green, positive density in the *F*_obs_ − *F*_calc_ electron density map) is visible close to the iron atom, indicating a reactive hydrogen peroxide species within the active site, but the resolution of the electron density map does not allow an atomic model to be built without additional chemical restraints. To address this issue, off-resonance Raman spectra were recorded from both crystals and solutions of superoxide reductase treated with hydrogen peroxide (Fig. 2*b* ▶). Two new Raman scattering peaks appear in these spectra at 567 and 840 cm^−1^, corresponding to iron–oxygen and oxygen–oxygen vibration of the iron peroxide species, respectively. With this additional spectroscopic information the active site was modelled to reflect the characteristic geometry of the iron peroxide intermediate.

For photoactive proteins, the reaction can be induced by light, and a large number of light-dependent proteins have been structurally characterized using low-temperature intermediate trapping methods. These studies include low-temperature trapping experiments on crystals of myoglobin:carbon monoxide complexes (Chu *et al.*, 2000 ▶; Ostermann *et al.*, 2000 ▶; Brunori *et al.*, 2000 ▶; Schlichting *et al.*, 1994 ▶; Teng *et al.*, 1994 ▶), photoactive yellow protein (Genick *et al.*, 1998 ▶), bacteriorhodopsin (Edman *et al.*, 1999 ▶; Royant *et al.*, 2000 ▶; Sass *et al.*, 2000 ▶; Luecke *et al.*, 1999 ▶, 2000 ▶), sensory rhodopsin II (Edman *et al.*, 2002 ▶; Moukhametzianov *et al.*, 2006 ▶), a photosynthetic reaction centre (Katona *et al.*, 2005 ▶; Stowell *et al.*, 1997 ▶) and a photoactivatable fluorescent protein (Adam *et al.*, 2008 ▶). Although a comprehensive treatment of each of these examples is beyond the scope of this review, it is noteworthy that the low-temperature intermediate trapping studies of phototriggered myoglobin in complex with carbon monoxide (Chu *et al.*, 2000 ▶; Schlichting *et al.*, 1994 ▶) drew structural conclusions similar to those recovered from later time-resolved Laue diffraction studies (Fig. 1 ▶). In particular, the locations of the transient binding pockets for carbon monoxide near the haem group (Figs. 1*c* ▶ and *d*) show very good agreement between the low-temperature structure (Chu *et al.*, 2000 ▶) and time-resolved Laue diffraction studies (Schotte *et al.*, 2003 ▶) (Figs. 1*a* and 1*b* ▶). This explicit agreement demonstrates the complementary nature of the two approaches, for which accurate structural information may be more rapidly forthcoming using low-temperature approaches, whereas information on the timescales of conformational changes can only be extracted using room-temperature time-resolved Laue diffraction methods. Moreover, helices E and F appear to undergo larger movements in those structures determined by time-resolved Laue crystallography when compared with those determined using cryo-trapping methods.

**Figure 3:**
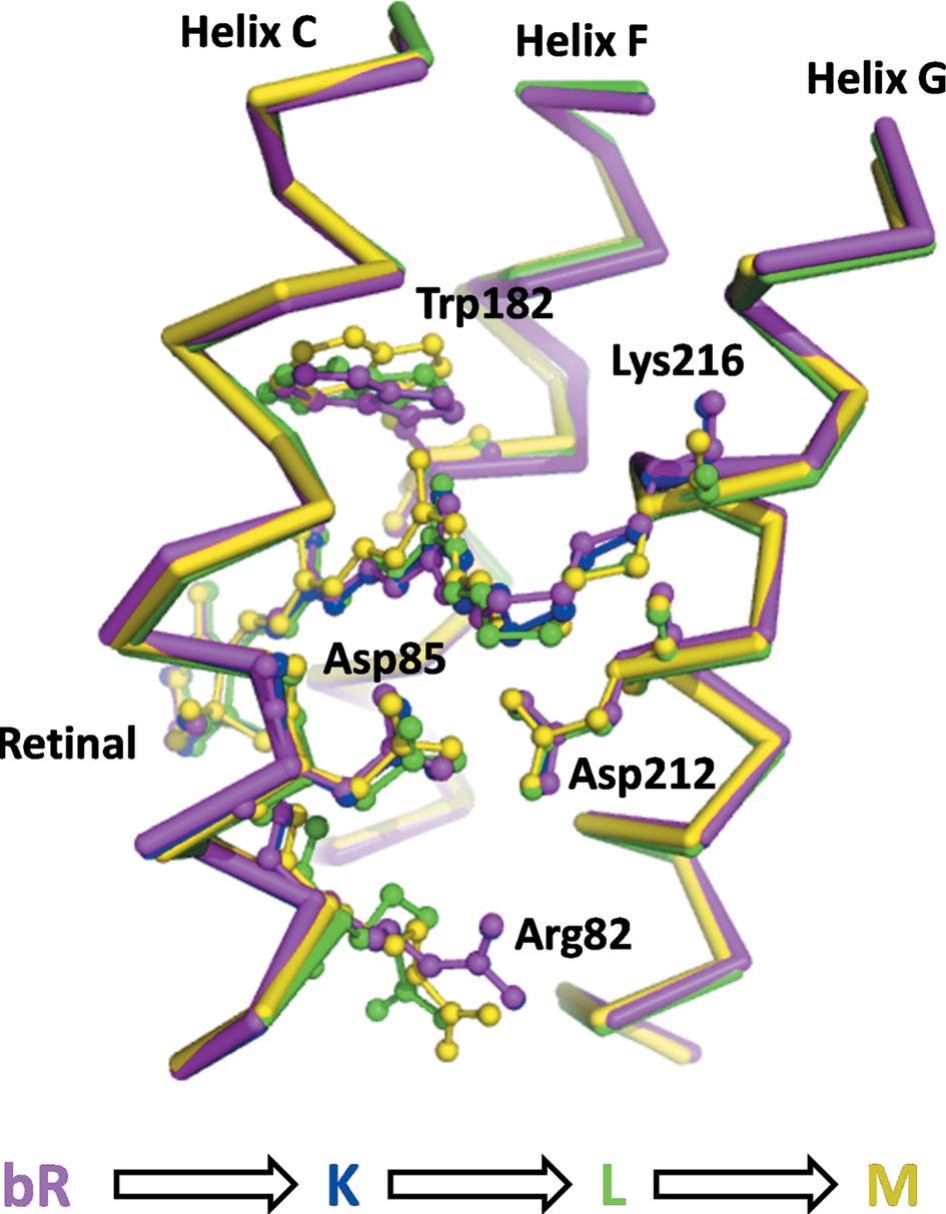
Structural results from intermediate trapping studies of bacteriorhodopsin. Four structures of resting (Belrhali *et al.*, 1999 ▶) (purple, Protein Data Bank entry 1qhj), early (Edman *et al.*, 1999 ▶) (blue, 1qkp), intermediate (Royant *et al.*, 2000 ▶) (green, 1eop) and late (Luecke *et al.*, 1999 ▶) (yellow, 1c8s) conformations are shown. These intermediate conformations were trapped by illuminating crystals at 110 K, 170 K and during thawing, respectively. A clear evolution of the retinal can be observed for these structures, which moves towards the cytoplasm as the temperature is raised. Moreover, significant displacements of Trp-182, Asp-85 and Arg-82 are also observed, as are rearrangements of water molecules recorded in the corresponding Protein Data Bank entries (not shown for reasons of clarity).

As a final example in this section we briefly review the structural results to emerge from intermediate trapping studies of bacteriorhodopsin, a seven-transmembrane integral membrane protein. This simplest known light-driven proton pump contains a buried all-*trans* retinal chromophore which is isomerized to its 13-*cis* conformation upon the absorption of a single photon. Retinal isomerization triggers a cascade of structural changes which ultimately lead to the vectorial transport of a proton ‘uphill’ against a *trans*-membrane proton gradient (Neutze *et al.*, 2002 ▶). The energy stored across this energy-transducing membrane is harvested by ATP-synthase (Stock *et al.*, 1999 ▶) to regenerate ATP, the basic energy currency of the cell. Fig. 3 ▶ illustrates the structural information that emerged from the first intermediate trapping studies on crystals of bacteriorhodopsin, for which monochromatic X-ray diffraction data were collected following illumination either at low temperature (Edman *et al.*, 1999 ▶; Royant *et al.*, 2000 ▶) or during thawing prior to flash-freezing (Luecke *et al.*, 1999 ▶). What was apparent from these structural results is a structural evolution of the retinal chromophore towards the cytoplasmic side of the protein, significant rearrangements of some key amino-acid side chains and water molecules (not illustrated) on the extracellular side of the protein, and indications of initial global rearrangements of α-helices. From these high-resolution structural results, in combination with an electron diffraction structure of a triple-mutant analogue of a late conformational state of bacteriorhodopsin (Subramaniam & Henderson, 2000 ▶), a coherent picture of the structural mechanism of proton pumping by bacteriorhodopsin rapidly emerged (Neutze *et al.*, 2002 ▶).

Several follow-up intermediate trapping studies were later performed, again using low-temperature illumination or thaw/freeze trapping protocols, and there are currently 20 entries in the Protein Data Bank pertaining to light (or mutation analogue)-induced conformational changes in bacterio­rhodopsin [see Hirai & Subramaniam (2009 ▶) and Andersson *et al.* (2009 ▶) for overviews]. Although the basic nature of the conformational changes occurring in the bacteriorhodopsin photocycle, such as retinal isomerization and water molecule movements, have been reproducibly observed, this set of structures (Edman *et al.*, 1999 ▶, 2004 ▶; Matsui *et al.*, 2002 ▶; Schobert *et al.*, 2002 ▶; Royant *et al.*, 2000 ▶; Lanyi & Schobert, 2002 ▶, 2003 ▶, 2006 ▶, 2007 ▶; Kouyama *et al.*, 2004 ▶; Luecke *et al.*, 1999 ▶, 2000 ▶; Sass *et al.*, 2000 ▶; Facciotti *et al.*, 2001 ▶; Schobert *et al.*, 2003 ▶; Takeda *et al.*, 2004 ▶; Subramaniam & Henderson, 2000 ▶; Rouhani *et al.*, 2001 ▶) disagree regarding the apparent timing of conformational changes and the magnitude of helical movements. These controversies illustrate a major short-coming of the intermediate trapping approach, since one can very accurately refine a protein structure against diffraction data, yet the structural conclusions may not be consistent with other structural studies. Moreover, when large-scale helical movements do occur, they unavoidably clash with, and hence potentially disrupt, the three-dimensional crystal lattice. As such, the natural tendency to focus on the highest-resolution highest-quality X-ray diffraction data may tend to screen for experiments in which the reaction was not successfully initiated at high concentration within crystals. In this context the quality of the experimental difference Fourier electron density map is an essential indicator of success. Irrespectively, should the crystal lattice inhibit large-scale helical movements then it will be impossible, even in principle, to reliably observe such movements using intermediate trapping or time-resolved Laue diffraction protocols. Thus, while intermediate trapping has proven to be a powerful and popular method for observing the structural details of how a reaction proceeds, other approaches are required to complement this detailed structural information.

## 4. Time-resolved wide-angle X-ray scattering

**Figure 4:**
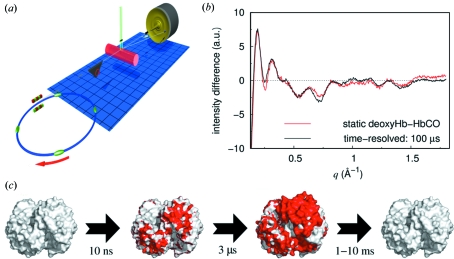
Time-resolved wide-angle X-ray scattering of the haemoglobin:carbon monoxide complex. (*a*) Sketch of the experimental set-up at the dedicated time-resolved beamline at the European Synchrotron Radiation Facility. Polychromatic X-ray pulses are generated in an undulator and a rotating chopper (triangle) is used to isolate a chosen pulse train. Protein samples are held within a glass capillary (red) and are excited by laser pulses (green) incident perpendicular to the X-ray beam. Concentric diffusive X-ray scattering rings are collected on a charge-couple device (CCD) detector. (*b*) Integration in rings and subtraction of ‘laser off’ images from ‘laser on’ images yields difference scattering curves, which are the fingerprint of the structural rearrangements in the protein. Difference scattering recorded from haemoglobin in complex with carbon monoxide 100 µs after photoexcitation (red) are compared with ‘static’ differences between haemoglobin with carbon monoxide bound and deoxyhaemoglobin (black). (*c*) Surface representation of the expected time-dependent structural changes in haemoglobin. Regions of the proteins that are involved in the changes are coloured red. Reproduced by permission from Macmillan Publishers: *Nature Methods* (Cammarata *et al.*, 2008 ▶), copyright (2008).

Although time-resolved Laue diffraction and intermediate trapping studies have provided several detailed insights into protein-mediated reactions, these methods necessarily probe protein conformational dynamics within the crystalline state. Structural probes applicable to liquid phases would explicitly side-step this fundamental limitation of time-resolved crystallographic studies of proteins. Time-resolved wide-angle X-ray scattering is an emerging technique that addresses this shortcoming, whereby X-ray scattering data are recorded as a function of time from an ensemble of samples in a liquid environment. Thus the breakage and formation of chemical bonds within small molecules, or the rearrangement of secondary structural elements within proteins, can be visualized since the internal distances between the atoms of the sample change with time. On the other hand, all structural information accessible using wide-angle X-ray scattering is averaged over all orientations of a randomly ordered ensemble of molecules within the sample. As such, the level of structural detail that can be envisioned, even in principle, is significantly less than what can be gleaned using X-ray diffraction. The experimental set-up at the ESRF is shown in Fig. 4 ▶(*a*).

Time-resolved wide-angle X-ray scattering was first successfully applied to probe the structural dynamics of a number of photosensitive small molecules in solution (Neutze *et al.*, 2001 ▶; Plech *et al.*, 2004 ▶; Davidsson *et al.*, 2005 ▶; Ihee, Lorenc * et al.*, 2005 ▶), all of which contained one or more heavy atoms such as iodine. The presence of heavy atoms within the photochemical of study was an essential ingredient since the challenge was to reliably extract a transient structural signal from a low-concentration photochemical intermediate of interest when, at the same time, the surrounding solvent molecules become heated and thereby also cause transient changes in the X-ray scattering data (Cammarata *et al.*, 2006 ▶; Georgiou *et al.*, 2006 ▶). For time-resolved X-ray scattering studies of small molecules in solution this problem has proven tractable, and several studies have unambiguously observed the transient conformations of short-lived photochemical systems in solution (Plech *et al.*, 2004 ▶; Davidsson *et al.*, 2005 ▶; Ihee, Lorenc *et al.*, 2005 ▶: Vincent *et al.*, 2009 ▶; Kong *et al.*, 2006 ▶, 2007 ▶, 2008 ▶; Lee, Kim, Cammarata *et al.*, 2008 ▶; Lee, Kim, Kim *et al.*, 2008 ▶).

Time-resolved wide-angle X-ray scattering has also recently been extended to probe the conformational dynamics of proteins (Cammarata *et al.*, 2008 ▶; Andersson *et al.*, 2009 ▶). This approach can be viewed either as an extension of the small molecule studies above to more complex systems, or as a higher-resolution extension of the method of time-resolved small-angle X-ray scattering, which provides low-resolution structural information on the dynamics of protein and RNA folding and oligomeric assembly (Lamb, Kwok *et al.*, 2008 ▶; Lamb, Zoltowski *et al.*, 2008 ▶; Wu *et al.*, 2008 ▶; Canady *et al.*, 2001 ▶; Russell *et al.*, 2000 ▶; Segel *et al.*, 1999 ▶). As a proof-of-principle demonstration, time-resolved wide-angle X-ray scattering data from haemoglobin in complex with carbon monoxide was studied (Cammarata *et al.*, 2008 ▶). Haemoglobin is the oxygen carrier of the blood, being present at extremely high concentrations in red blood cells and giving them their strong red colour. This tetrameric complex contains two copies of its α- and β-subunits, both of which follow the myoglobin α-helical fold surrounding an active-site haem group. As with time-resolved diffraction studies of myoglobin (Fig. 1 ▶), laser photolysis of haemoglobin in complex with carbon monoxide dislodges the haem ligand and allows the protein to relax. In contrast with myoglobin, however, this active-site disruption triggers a global rearrangement of the entire multi-subunit complex, as illustrated in Fig. 4(*c*) ▶. These large-scale conformational changes have been studied extensively and provide the best understood example of protein cooperativity between different subunits of a multi-domain protein (Perutz *et al.*, 1998 ▶). In haemoglobin, structurally mediated cooperative effects fine-tune the protein’s oxygen affinity in the lungs and blood vessels.

Difference time-resolved wide-angle X-ray scattering data recorded from solubilized haemoglobin are reproduced in Fig. 4(*b*) ▶. As demonstrated by Cammarata *et al.* (2008 ▶), the position and amplitudes of the oscillations in the time-resolved X-ray scattering data recorded 100 µs following photoactivation (Fig. 4*b* ▶, black line) correlated well with differences in scattering from static wide-angle X-ray scattering measurements of deoxyhaemoglobin and haemoglobin in complex with carbon monoxide (Fig. 4*b* ▶, red line). Moreover, the timescales of these conformational changes could be observed, with a very rapid initial protein conformational response being recorded already after 200 ns. This was followed by the larger global conformational rearrangements illustrated in Fig. 4(*c*) ▶, which arose on a timescale of microseconds and decayed within milliseconds. What was apparent from this study was that haemoglobin, within this liquid environment and therefore unconstrained by crystal lattice contacts, underwent precisely those global rearrangements that have been characterized through static X-ray crystallographic structures of the oxy- and deoxyhaemoglobin forms.

Similar studies of the photolysis of carbon monoxide:myoglobin complexes have revealed somewhat larger conformational changes associated with helix F in solution, rather than in the crystalline state (Ahn *et al.*, 2009 ▶). As with an earlier time-resolved small-angle X-ray scattering study (Segel *et al.*, 1999 ▶), Cammarata *et al.* (2008 ▶) also demonstrated the principle that protein folding events in cytochrome-c could be followed by time-resolved wide-angle X-ray scattering. In this case cytochrome-c was partially unfolded with a denaturing agent enabling carbon monoxide to bind. When a laser flash was again used to disrupt the binding of carbon monoxide to the haem group, the protein spontaneously folded, and a difference wide-angle X-ray scattering signal from this event was resolved with time.

**Figure 5:**
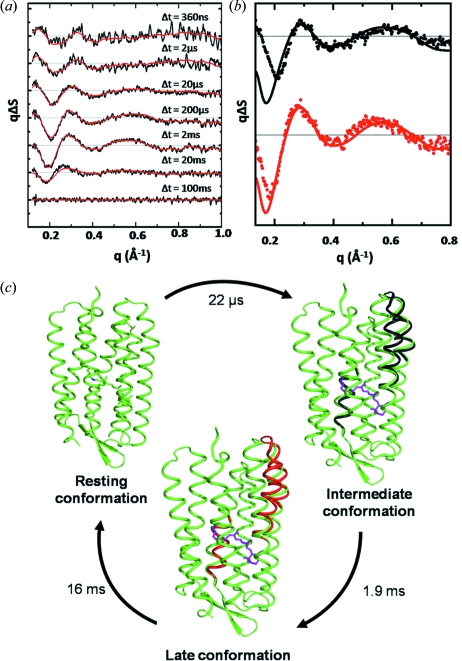
Time-resolved wide-angle X-ray scattering data from solubilized samples of bacteriorhodopsin. (*a*) Integration in rings and subtraction of ‘laser off’ images from ‘laser on’ images yields the difference scattering curves as a function of the time delay (Δ*t*) between photoactivation and the X-ray probe. (*b*) Basis spectra of an intermediate and late conformational state extracted from spectral decomposition of the data shown in (*a*). (*c*) Structural interpretation of these data using a rigid-body minimization routine illustrated in terms of the bacteriorhodopsin photocycle (Andersson *et al.*, 2009 ▶). Reproduced from *Structure* (Andersson *et al.*, 2009 ▶), copyright (2009), with permission from Elsevier.

Time-resolved wide-angle X-ray scattering has also been used to probe the timescales and magnitudes of α-helical movements associated with the photocycles of the light-driven proton pump bacteriorhodopsin, and a close relative proteorhodopsin from photosynthetic oceanic bacteria (Andersson *et al.*, 2009 ▶). Unlike haemoglobin, however, the nature, extent and timing of secondary structural element rearrangements within the photocycle of bacteriorhodopsin have been controversial (Hirai & Subramaniam, 2009 ▶; Andersson *et al.*, 2009 ▶). Fig. 5(*a*) ▶ illustrates the time-resolved wide-angle X-ray scattering difference data recorded from bacteriorhodopsin as a function of the time delay, Δ*t*, following photoactivation. These data were fitted to three basis spectra and their characteristic time constants determined. Structural refinement against the intermediate and late state basis spectra (Fig. 5*b* ▶), which have population maxima at approximately 60 µs and 5 ms following photoactivation, concluded that a significant outwards movement of helices E and F occurred on the cytoplasmic half of the protein concomitant with an inwards flex of helix C on the extracellular side (Fig. 5*c* ▶). While these major structural conclusions were consistent with findings from light-induced movements in three-dimensional crystals of bacteriorhodopsin trapped at low temperature (Royant *et al.*, 2000 ▶; Edman *et al.*, 2004 ▶) and mutation-induced conformational changes observed by two-dimensional electron crystallography (Subramaniam & Henderson, 2000 ▶), it is significant that the magnitudes of these motions for detergent solubilized protein were significantly larger than those motions observed in the crystalline state. Moreover, the conformational state adopted by bacteriorhodopsin after 60 µs showed approximately two-thirds of the helical motions associated with the late conformational state, establishing that significant movements of α-helices arise prior to the primary proton transfer event of the bacteriorhodopsin photocycle (Andersson *et al.*, 2009 ▶). Structural refinement against the difference data recorded from proteorhodopsin drew similar conclusions as to the nature and magnitudes of the helical movements, providing direct experimental evidence that the basic structural mechanism of proton pumping by bacteriorhodopsin is preserved across widely evolutionary divergent species.

These results show that high-quality transient difference X-ray scattering data can be recovered from proteins in solution, and a structural interpretation consistent with the known conformational dynamics can be drawn. A major plus for this developing approach is that it provides a direct measure of the timescales of protein conformational changes which, at best, can otherwise be acquired only indirectly through spectroscopic techniques. Nevertheless, the level of structural detail which can be extracted from the basis spectra (Fig. 5*b* ▶) is limited to a relatively low-resolution average over random orientations of the protein ensemble, and hence high-resolution insights into the specific structural events driving key chemical steps cannot be derived. Moreover, a significant number of theoretical challenges will have to be addressed in order to accurately and uniquely refine those conformational changes that occur. This is especially pertinent when studying other proteins for which less structural information is accessible *a priori* than for the pioneering examples of haemoglobin (Cammarata *et al.*, 2008 ▶), myoglobin (Ahn *et al.*, 2009 ▶) and bacteriorhodopsin (Andersson *et al.*, 2009 ▶). For membrane transport proteins it has been proposed that, by labelling the substrate and transport channel with heavy-atom markers, it may be possible to extend this method to provide an experimental probe capable of simultaneously resolving both local and global conformational changes (Andersson *et al.*, 2008 ▶). Nevertheless, for most systems of interest it will certainly be necessary to collect additional high-resolution structural information using the tools of Laue diffraction, intermediate trapping or X-ray spectroscopic techniques, in order to piece together an overall structural mechanism of action.

## 5. Time-resolved X-ray absorption spectroscopy

Another solution-based approach for probing protein structural dynamics is to use time-resolved X-ray absorption or fluorescence techniques. These spectroscopic methods are well suited for characterizing the coordination structure of active sites centres of metalloproteins (Strange *et al.*, 2005 ▶; Penner-Hahn, 2005 ▶; Levina *et al.*, 2005 ▶; Strange & Feiters, 2008 ▶). An elegant aspect of X-ray spectroscopy is that the metal centre of interest functions as both the source and the detector of its local chemical environment. Specifically, tunable monochromatic X-rays from a synchrotron light source are used to excite the core electrons of the absorbing atom to be probed. As these core electrons are excited into the continuum and ejected from the parent atom, the resulting photoelectron wave is scattered by the neighbouring atoms. This scattering in turn interferes with the wavefunction of the emitted photoelectron, and interference effects thus alter the absorption and fluorescent properties of the metal centre in a manner which depends explicitly upon its local geometry. Thus, by recording changes in the X-ray spectra with time it is possible to track geometrical changes in the local vicinity of the metal centre (Bressler *et al.*, 2008 ▶).

**Figure 6:**
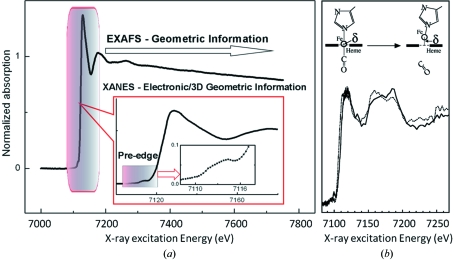
X-ray absorption spectra from iron-containing proteins. (*a*) Static X-ray absorption spectra from the iron *K* edge of PerR protein (Jacquamet *et al.*, 2009 ▶). Extended X-ray absorption fine structure (EXAFS) records the modulation of an X-ray absorption spectrum in the energy region from 50 eV to approximately 1000 eV above the absorption edge, providing structural information on the neighbours of the absorbing atom and very accurate first-shell iron–ligand distances. X-ray absorption near-edge structure (XANES) focuses upon the smaller energy region up to approximately 50 eV above the edge and provides structural and electronic information for the absorbing atom. The pre-edge region of XANES is sensitive to the oxidation, spin state and geometric environment of the absorbing atom. (*b*) Time-resolved X-ray absorption spectra of myoglobin in complex with carbon monoxide and its interpretation in terms of structure. Iron *K*-edge XANES spectra (dashed line) were recorded 100 µs following photoexcitation, and spectra from the resting conformation are shown for comparison (black line). Spectral changes were interpreted as resulting from the movement of carbon monoxide away from the haem group (inset). Reprinted from *Journal of Electron Spectroscopy and Related Phenomena* (Wang *et al.*, 2005 ▶), copyright (2005), with permission from Elsevier.

Extended X-ray absorption fine structure (EXAFS) records the modulation of an X-ray absorption spectrum in the energy region from 50 eV to approximately 1000 eV above the absorption edge, whereas X-ray absorption near-edge structure (XANES) focuses upon the smaller energy region up to approximately 50 eV above the edge (Fig. 6*a* ▶). From the analysis of EXAFS it is possible to extract the number, type and distances to those atoms which surround the probed metal centre, whereas XANES provides information on the geometrical arrangement of the ligands in contact with the absorbing atom as well as its effective charge. Advanced fitting algorithms against XANES spectra allow structural information such as bond lengths and angles to be accurately refined (Benfatto *et al.*, 2001 ▶; Smolentsev & Soldatov, 2006 ▶; Sarangi *et al.*, 2008 ▶; Jacquamet *et al.*, 2009 ▶) although this comes with the caveat that a sufficiently accurate initial structural model is an essential starting point.

Pioneering studies using ultra-fast time-resolved XANES observed the transient formation of a charge-transfer excited state of photoexcited rubidium complexes with a lifetime of 300 ns (Saes *et al.*, 2003 ▶). The approach has also been successfully extended to probe rapid light-driven conformational changes about iron centres (Gawelda *et al.*, 2007 ▶), recently achieving a temporal resolution in the femtosecond regime (Bressler *et al.*, 2009 ▶), and also to probe rapid light-induced rearrangements in diplatinum molecules (van der Veen *et al.*, 2009 ▶). However, as with time-resolved wide-angle X-ray scattering, it remains a significant challenge to extend these impressive results for small molecules in solution to probe protein structural dynamics. The main limitations are practical rather than issues of principle, since it is frequently difficult to concentrate proteins to the m*M* concentrations required to recover good signal to noise in the X-ray spectra, and it is normally not realistic to make tens to hundreds of millilitres of highly concentrated sample.

Myoglobin in complex with carbon monoxide again provided a convenient system for proof-of-principle X-ray spectroscopy studies of protein conformational dynamics about a metal centre. It is more than two decades since the first time-resolved X-ray absorption near-edge spectra of the photodissociation of carbon monoxide from the haem iron of myoglobin were described (Mills *et al.*, 1984 ▶). In these pioneering studies changes in the pre-edge structure and in the position of the iron edge were detected as a function of time, and were interpreted in terms of the effective charge of the iron and changes in its coordination from 6 to 5 following photodissociation of carbon monoxide. More recent studies on the same system (Wang *et al.*, 2005 ▶) have improved the temporal resolution to 100 µs, and the XANES spectra obtained in that study are reproduced in Fig. 6(*b*) ▶. Only qualitative interpretation of the observed oscillations within the XANES were reported, and were based upon empirical correlations between the displacement of Fe atoms out of the haem plane and the relative intensities of spectral features (Wang *et al.*, 2005 ▶).

Another noteworthy time-resolved X-ray spectroscopy study on proteins is the observation of light-induced redox changes within the oxygen-evolving centre of photosystem II (Haumann *et al.*, 2005 ▶, 2008 ▶). This large membrane protein complex harvests light so as to transfer electrons across a photosynthetic energy-transducing membrane which, in combination with other coupled redox reactions, creates a transmembrane proton gradient. In the process, photosystem II extracts electrons from water and forms molecular oxygen as a by-product, a convenient choice of electron donor that ultimately changed the earth’s atmosphere and drove evolution. This remarkable chemistry occurs at the oxygen-evolving centre of photosystem II, which is formed by four manganese atoms and a single calcium atom (Ferreira *et al.*, 2004 ▶; Loll *et al.*, 2005 ▶; Guskov *et al.*, 2009 ▶). Polarized EXAFS measurements on both crystals (Yano *et al.*, 2006 ▶) and membrane preparations of photosystem II (Dau *et al.*, 2008 ▶) have been used to accurately constrain the geometry of the manganese cluster, which has been controversial owing to the X-ray-induced reduction of the manganese cluster in diffraction studies (Yano *et al.*, 2005 ▶). Furthermore, time-resolved traces of X-ray absorption amplitudes for photosystem II with a limited number of energies successfully monitored the light-induced change in redox states of the oxygen-evolving centre with a time resolution of 10 µs (Haumann *et al.*, 2005 ▶, 2008 ▶). From these studies, Haumann *et al.* have exploited this unique dynamical information to help characterize the time evolution of important redox changes within the manganese cluster during the oxygen-evolving reaction.

Although not yet widely applied as a tool to capture protein structural dynamics, time-resolved X-ray absorption spectroscopy appears to be a very promising method as a probe of local atomic movements near a protein’s active site. Moreover, a number of intermediate trapping studies on proteins have also been performed using X-ray absorption methods (Kleifeld *et al.*, 2001 ▶, 2003 ▶; Solomon *et al.*, 2007 ▶), including one low-temperature study on myoglobin in complex with small molecules (Saigo *et al.*, 1993 ▶). While several practical issues remain, with ongoing technical development we anticipate that the scope of application of these spectroscopic approaches for studying metal-containing proteins will grow. As such, this method holds promise in providing a potentially valuable complement to other emerging X-ray methods for piecing together a complete structural description of a protein’s reaction mechanism.

## 6. Future developments with synchrotron radiation

A smorgasbord of experimental approaches exploiting synchrotron-generated X-rays have emerged to probe protein structural dynamics. In this review we have outlined several achievements of time-resolved Laue diffraction, intermediate trapping techniques in three-dimensional crystals, time-resolved wide-angle X-ray scattering and X-ray absorption spectroscopy. It is our view that each of the methods offers valuable insight into the structural chemistry of biomolecular reactions, for which a high-resolution protein structure provides an initial starting point.

For time-resolved Laue diffraction the bottom line remains to continuously improve light-sensitive crystals to the point where they diffract to high resolution, have very low mosaic spread, and are resistant to damage from both laser photolysis and exposure to X-rays. For light-driven systems undergoing large-scale conformational changes, such as haemoglobin and bacteriorhodopsin (Figs. 4*c* ▶ and 5*c* ▶), the strain induced on the crystal lattice seems likely to prevent such motions being captured by Laue diffraction. On the other hand it is reasonable to expect that the list of proteins for which time-resolved Laue diffraction studies provide novel structural insight continues to grow, albeit slowly. Likewise, the field of intermediate trapping of reaction intermediates in three-dimensional crystals will continue to be applied to an increasingly broad group of proteins, since the technical demands and crystal quality, reaction triggering and reversibility associated with such studies are not at all as severe as for Laue diffraction.

Time-resolved wide-angle X-ray scattering is a less mature method, but holds tremendous promise by providing direct global structural information from proteins in a native-like phase. Since spectroscopic methods have indicated disagreements between protein rearrangements in the crystalline and solution phases (Heberle & Gensch, 2001 ▶), this structural probe will prove valuable by addressing head-on these concerns. For the field to gain wider acceptance, however, a theoretical challenge concerning the uniqueness of solutions that emerge from structural refinement against wide-angle X-ray scattering data (Andersson *et al.*, 2009 ▶) will have to be addressed. This concern will be especially pertinent for proteins for which no structural information concerning their reaction pathway is available *a priori* to guide structural analysis. Nevertheless, we are confident that synergies can be developed between time-resolved wide-angle X-ray scattering studies and existing computational methods such as coarse grain models (Stumpff-Kane *et al.*, 2008 ▶; Tozzini, 2005 ▶), normal-mode analysis of molecular dynamics trajectories (Stumpff-Kane *et al.*, 2008 ▶) and geometric sampling of protein conformational transitions (Seeliger *et al.*, 2007 ▶), so as to converge upon unique conformational states with a minimum number of additional structural assumptions. Moreover, variations of time-resolved wide-angle X-ray scattering which introduce heavy atoms or other strong scatterers into a protein (Andersson *et al.*, 2008 ▶) could be used to develop objective methods for uniquely specifying structural changes, in analogy with the use of heavy-atom derivatives for phasing purposes in X-ray crystallography.

Significant technical challenges will also be encountered when moving from light-driven and reversible proof-of-principle systems of study (Cammarata *et al.*, 2008 ▶; Andersson *et al.*, 2009 ▶) to structurally characterize the conformational dynamics of chemically driven irreversible reactions, but there are no reasons in principle why these challenges cannot be overcome. Promising options in this respect include developing micro-fluidics stopped-flow devices or the use of caged compounds to allow light activation of chemically driven reactions, with the latter technology being particularly well developed in other fields of biology (Ellis-Davies, 2007 ▶; Kao, 2006 ▶; Mayer & Heckel, 2006 ▶). Since most biological reactions are chemically triggered, the successful application of such strategies for time-resolved wide-angle X-ray scattering would pave the way for these experiments to become more widely applied.

Time-resolved X-ray absorption spectroscopy can also be expected to gain in popularity for applications on metal-containing proteins. In particular, the impressive recent progress in ultra-fast time-resolved XANES spectroscopy (Saes *et al.*, 2003 ▶; Gawelda *et al.*, 2007 ▶; Bressler *et al.*, 2009 ▶; van der Veen *et al.*, 2009 ▶) bodes well for overcoming technical challenges associated with dynamical studies of proteins on longer timescales. Nevertheless, significant practical challenges will remain since relatively few proteins can be concentrated to m*M* levels, creating signal-to-noise concerns that limit the attainable time resolution and data quality. An attractive foreseeable development with synchrotron-based X-ray absorption studies is the possibility of recording high-quality X-ray spectra from proteins using softer X-rays (Aziz *et al.*, 2009 ▶) and extending recent time-resolved soft X-ray absorption studies on small molecules in solution (Huse *et al.*, 2009 ▶) to macromolecules. In these cases it may be possible to follow with time changes in the *L* edges of metals, and potentially the *K* edge of lighter atoms, such as sulfur, could be probed. As these capabilities come online they will create new possibilities for time-resolved X-ray absorption spectroscopy methods to have impact in characterizing protein conformational changes about selected atoms.

## 7. Potential impact of an X-ray free-electron laser

Beyond what may be achievable by means of foreseeable developments at current synchrotron radiation sources, the next generation of X-ray facilities are likely to revolutionize time-resolved structural studies of proteins. Hard X-ray free-electron lasers (XFELs) are currently under construction in Europe (http://xfel.desy.de/) and Japan (http://www-xfel.spring8.or.jp/) and the first American XFEL facility has recently reported lasing at 8 keV (http://lcls.slac.stanford.edu/). These fourth-generation X-ray sources promise extremely intense hard X-ray bursts of approximately 100 fs in duration, and will thereby create new opportunities for imaging of biological molecules from extremely small samples (Neutze *et al.*, 2000 ▶, 2004 ▶).

Within the realm of time-resolved pump–probe structural studies, the most obvious benefit of these emerging sources will be their remarkable capability to probe the structural dynamics of light-driven reactions with a temporal resolution of 100 fs. Several landmark measurements have already been reported on this timescale at linear-accelerator-based X-ray sources, including key technical innovations for synchronizing the femtosecond laser and X-ray pulses (Cavalieri *et al.*, 2005 ▶), surface melting studies of semiconductor crystals following photoactivation by a femtosecond laser (Lindenberg *et al.*, 2008 ▶), and the development of a novel reflective experimental geometry to follow the X-ray-induced Coulomb explosion of small polystyrene spheres with femtosecond resolution (Chapman *et al.*, 2007 ▶). When XFEL-generated ultra-fast X-ray pulses are used to probe the structural dynamics of proteins with time, it will become possible, at least in principle, to follow the evolution of isomerization, bond breaking and charge-transfer reactions on the timescales at which these fundamental photochemical processes occur.

While taking a very optimistic viewpoint of these emerging opportunities, it seems likely that several technical challenges will be encountered along this path. For example, when using pump–probe methods to study protein reaction dynamics in the crystalline phase, it may well prove advantageous to use the full width of the background undulator spectrum of the XFEL to perform ‘pink’ time-resolved Laue-diffraction (Bourgeois *et al.*, 2003 ▶, 2006 ▶; Schotte *et al.*, 2003 ▶, 2004 ▶; Anderson *et al.*, 2004 ▶; Rajagopal *et al.*, 2005 ▶; Ihee, Rajagopal *et al.*, 2005 ▶; Schmidt *et al.*, 2004 ▶) rather than exploit the narrow extremely brilliant lasing line of the free-electron laser fundamental. On the other hand, the remarkable peak brilliance of the XFEL fundamental will enable interpretable X-ray diffraction patterns to be recorded from nanometre-scale crystals a few unit cells across (Neutze *et al.*, 2000 ▶, 2004 ▶), and one can imagine pump–probe time-resolved X-ray diffraction studies being performed from a sequence of nanocrystals, each of which is exposed only once, whereupon it is destroyed by a highly focused XFEL beam (Neutze *et al.*, 2000 ▶). Such an approach would potentially facilitate extremely rapid chemical triggering of reactions within nanocrystals by rapid mixing, and thereby escape the limited set of light-triggered systems of study, but would necessarily involve many other technical challenges associated with processing and scaling of X-ray diffraction data from a random ensemble of nanocrystals.

Time-resolved wide-angle X-ray scattering (Cammarata *et al.*, 2008 ▶; Andersson *et al.*, 2009 ▶) and X-ray absorption studies (Wang *et al.*, 2005 ▶) in the femtosecond time domain will also be possible for protein samples at an XFEL. On this timescale, however, the protein movements are likely to be small, increasing the already severe demands on signal-to-noise ratio and detector stability. Nevertheless, for timescales faster than the time required for rotational redistribution, protein molecules will be preferentially photoexcited according to the orientation of their dipole moment, and this preferred alignment will be reflected as additional information in the X-ray scattering pattern. When interpreting time-resolved wide-angle X-ray scattering data, one further issue will arise on the sub-picosecond timescale, since heat that is deposited into the liquid sample by short laser pulses (Cammarata *et al.*, 2006 ▶; Georgiou *et al.*, 2006 ▶) will not have time to equilibrate before the arrival of the X-ray probe. Thus the presence of ‘hot spots’ in the surrounding solvent will be reflected in the X-ray scattering difference data, and approaches will need to be developed to handle this phenomenon. For time-resolved X-ray absorption spectroscopy, for which the structural data are necessarily confined to the local environment of the metal centre of interest, a non-equilibrated response of the solvent owing to rapid heating should not, in principle, pose new interpretational challenges.

In closing, the last decade has witnessed several significant technical improvements to existing methods, as well as the development of novel approaches, for elucidating the structural details of protein catalyzed biochemical reactions as a function of time. Even a conservative extrapolation of these recent developments can foresee intermediate trapping and time-resolved wide-angle X-ray scattering studies becoming increasingly widely applied approaches within structural biology. We also foresee that that domain of applications of time-resolved Laue diffraction and X-ray absorption spectroscopy will grow, albeit more slowly. Finally, the imminent application of ultra-fast extremely intense XFEL-generated X-ray pulses to probe the reaction pathways of light-sensitive proteins will offer unique opportunities, delivering many surprises as it opens new structural windows upon fundamental photochemical events on the timescale at which they occur.
